# Supplementary figures and images for: Spatial Transcriptomics Reveals Signatures of Histopathological Changes in Muscular Sarcoidosis
Source: Cells. 2023 Nov 30;12(23):2747. doi: 10.3390/cells12232747 (PMC10706822; doi:10.3390/cells12232747)

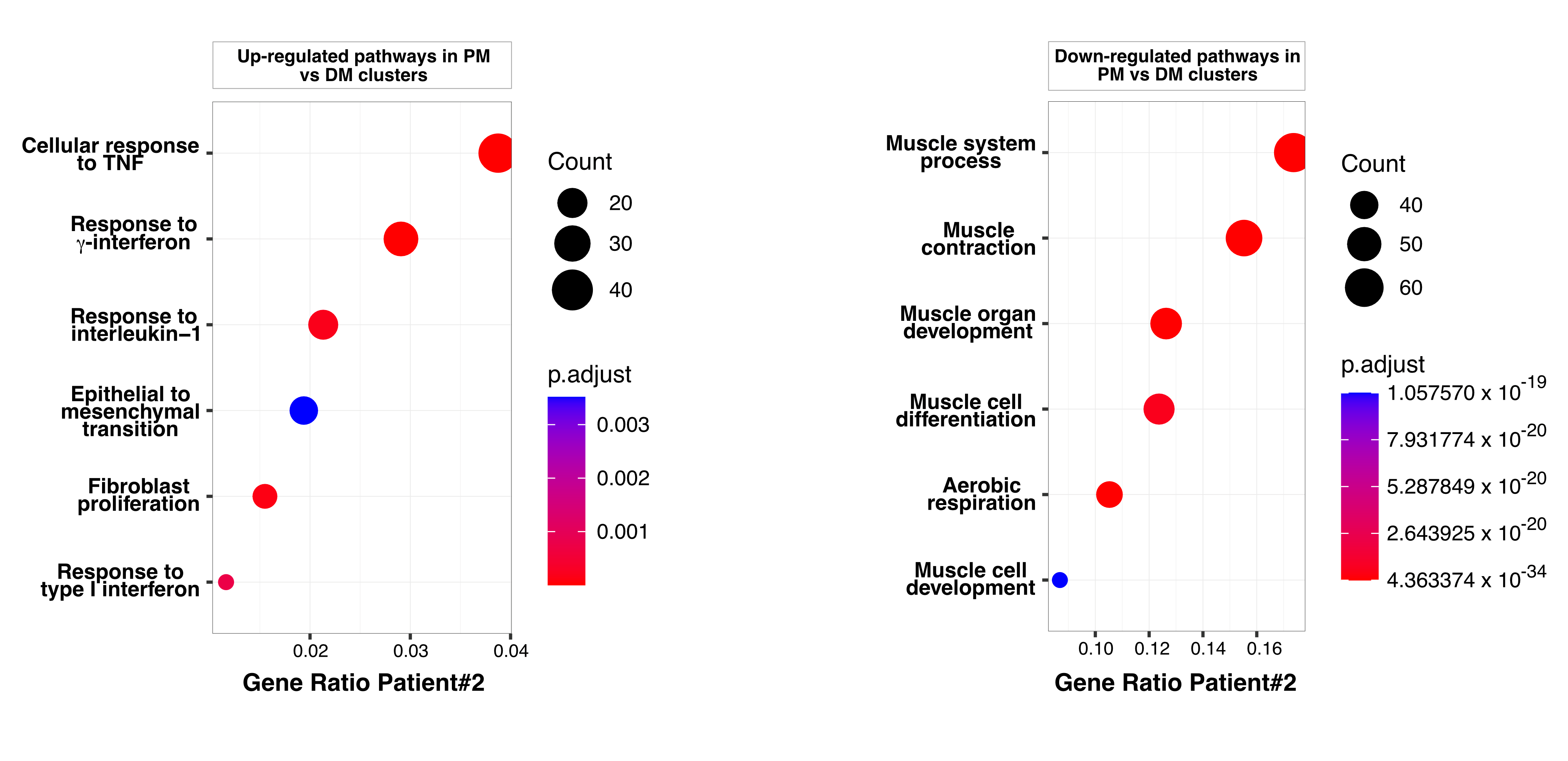

Supplement: Supplementary file 1 [file cells-12-02747-s001.zip › LEQUAIN-Figure-S2-CELLS-HighResol.tiff]

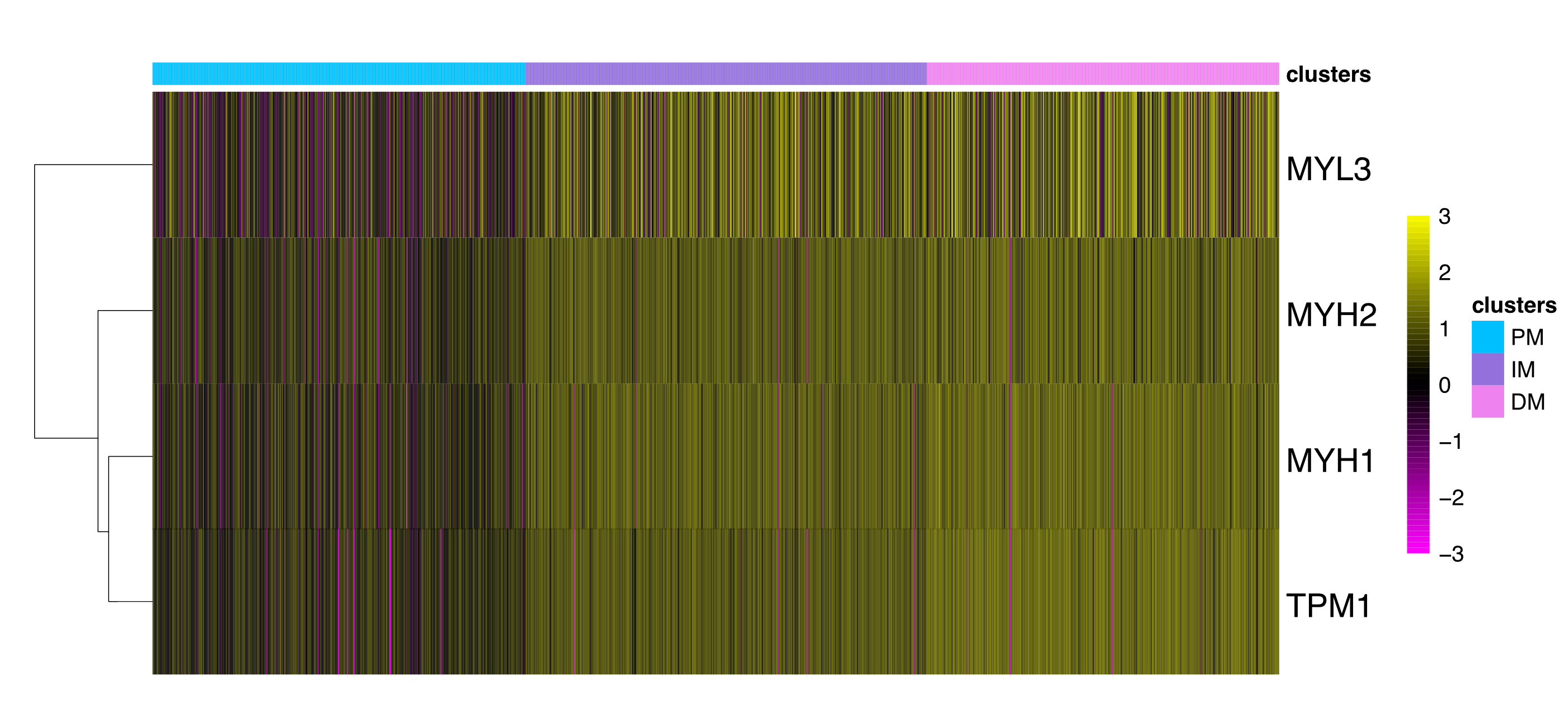

Supplement: Supplementary file 1 [file cells-12-02747-s001.zip › LEQUAIN-Figure-S3-CELLS-HighResol.tiff]
